# Development and Evaluation of a Slow-Release Occluded Fertilizer Employing Functionalized Biosolids as a Support Matrix

**DOI:** 10.3390/plants14203154

**Published:** 2025-10-13

**Authors:** Rodrigo Ramírez Palacios, Nora Restrepo-Sánchez, Rosember Ramirez, Isabel Acevedo Restrepo, Carlos Peláez Jaramillo

**Affiliations:** 1 Interdisciplinary Group of Molecular Studies (GIEM), Chemistry Institute, Faculty of Exact and Natural Sciences, University of Antioquia, Street 67 No. 53-108, Medellín 050010, Colombia; 2 Department of Engineering, Technological University of Chocó, Carrera 22 No. 18B-10, Quibdó 270002, Colombia

**Keywords:** slow release fertilizer, organic matter, clay, biosolid, paper sludge

## Abstract

In this study, a slow-release fertilizer (SRF) was obtained by occluding NPK 10–10–10 into two matrices and compared with the uncoated mineral fertilizer (F). The first matrix, FOMI, used biosolids/paper sludge at 3:1 (*w*/*w*); the second, FOMII, used biosolids/clay at 1:1 (*w*/*w*). Materials and pellets were physiochemically and microbiologically characterized. Release kinetics were evaluated in water and in soil columns packed with acid-washed sand; matrix-only controls and sand blanks confirmed negligible background N, P, and K. The uncoated mineral fertilizer (F) showed a rapid burst, whereas occlusion slowed release. FOMII reduced release relative to F, and FOMI produced the slowest, controlled profiles: kinetic fits yielded lower k values for FOMI than for FOMII and F. FOMI also exhibited higher water-retention capacity (WRC) and cation-exchange capacity (CEC), consistent with its greater organic-matter content. In soil, FOMI released less than 15% at 48 h and no more than 75% at 30 d, meeting European Committee for Standardization (CEN) SRF criteria; FOMII released faster than FOMI but slower than F, which exceeded 90% within the test period. Therefore, FOMI is a biodegradable, low-cost SRF that improves fertilizer-use efficiency while returning organic matter to agricultural soils; FOMII shows intermediate yet beneficial performance.

## 1. Introduction

Feeding a growing population amid persistent pressures—climate change, water scarcity, soil degradation, nutrient imbalances, shrinking arable land, and suboptimal agronomic practices—remains a defining challenge for global food security. Recent syntheses show that nutrient imbalances and declining soil fertility jeopardize yields, degrade ecosystem services, and elevate human-health risks via eutrophication and contamination of drinking water [1,2]. Concomitant declines in soil biodiversity and fertility are linked to reduced nutritional quality of foods, underscoring the urgency of soil-health restoration [3]. At the same time, nitrogen use efficiency (NUE) remains modest (global pooled ≈ 48–55%), meaning that large fractions of applied N are lost to the environment; poor synchronization between supply and crop uptake can push losses to 85–90% [4,5]

Mineral NPK fertilizers are indispensable inputs for sustaining crop performance [6], yet they do not replenish soil organic matter (OM)—which is critical for microbial function—and can generate environmental externalities [7]. Agronomic and climatic factors often drive substantial losses via ammonia volatilization, runoff, and leaching [8,9]. Field studies continue to document significant NH_3_ emissions and runoff-driven N and P export—key drivers of eutrophication and greenhouse-gas emissions—while confirming that improved practices (e.g., moderated rates and soil incorporation) can mitigate these fluxes [10,11,12,13].

Slow-release fertilizers (SRFs) aim to synchronize nutrient supply with plant demand, reducing application frequency and off-site losses [14]. Two broad approaches prevail: intrinsically low-solubility salts and encapsulated/coated fertilizers, with most commercial SRFs in the latter category [15]. Coatings often employ synthetic polymers (e.g., polysulfone, PVC, polystyrene, acrylamide, alginate composites), which can be costly and non-biodegradable [16,17,18]. Mounting assessments warn that polymer-coated fertilizers may contribute microplastics to agroecosystems—on the order of thousands of tons annually in Europe— underscoring the need for biodegradable carriers [19,20,21].

Biodegradable organo-mineral matrices are promising alternatives because they couple water-retention capacity (WRC) and cation-exchange capacity (CEC) with sorption sites that modulate nutrient transport. OM carries negatively charged functional groups that form oxidized complexes with cations [22], while clays provide permanent negative charge via isomorphic substitution [23]. These features underpin diffuse double-layer formation and ion adsorption [24], increasing WRC, CEC, and buffering capacity [25,26], thereby reducing leaching [14,27,28]. Recent evidence shows that higher soil organic carbon and improved aggregate stability enhance water and nutrient holding and microbial biomass—key mechanisms behind reduced nutrient losses—whereas the permanent charge of clay minerals strengthens cation retention [29,30,31]. In parallel, hydrogel/biopolymer systems (e.g., starch–alginate) are emerging as sustainable carriers that also enhance water retention capacity [32,33,34].

Within this context, we evaluated a slow-release strategy that occluded mineral salts (urea, diammonium phosphate, and potassium chloride) within high-OM matrices (the full treatment set and dosing scheme are provided in Table 1). We selected biosolids as the primary carriers given their OM content, WRC, and CEC, which can synergistically delay nutrient diffusion while valorizing residual streams [35,36,37]. Because biosolids contain endogenous N and P, we (i) quantified TKN and total P of the biosolid (Table 2), (ii) accounted for these contributions in the nominal 10–10–10 mass balance, and (iii) ran matrix-only controls to estimate any co-release of endogenous nutrients. Endogenous N and P in biosolids are largely associated with organic/particulate fractions and exchange sites, and are therefore mobilized more slowly than the occluded mineral salts [38,39,40]. Complementary carriers include paper-sludge cellulose and bentonite clay, selected for hydrophilicity/CEC and absorptive properties, respectively; sourcing and unit operations are detailed in Section 2.

To ensure safety and compliance, metals in biosolids and pellets were benchmarked against NTC 5167 [41] and EU 2019/1009 limits [42], and microbiological criteria were verified. Regarding emerging contaminants, recent literature indicates that biosolids may carry trace organic compounds and microplastics; rigorous deployment should therefore include source control, stabilization/sanitization verification, and fate/leaching tests [31,43]. While these aspects are beyond the scope of our kinetic focus, we flag them as prerequisites for field application and long-term monitoring.

Here, our objective was to quantify water- and soil-release kinetics of N, P, and K from occluded pellets relative to uncoated fertilizer, and to test the hypothesis that high-OM/CEC matrices reduce apparent rate constants and cumulative release through swelling- and sorption-controlled transport. We further interrogated mechanistic underpinnings via kinetic modeling, linking material properties to release profiles, with the overarching goal of advancing low-cost, biodegradable SRFs that improve fertilizer-use efficiency and soil health.

## 2. Materials and Methods

### 2.1. Reagents and Support Materials

The formulation of fertilizers was carried out with technical-grade reagents: urea 46% N (CO(NH_2_)_2_; Merck KGaA, Darmstadt, Germany), DAP 46% P and 18% N ((NH_4_)_2_HPO_4_; Delcorp, and potassium chloride (KCl) 60% K (Merck KGaA, Darmstadt, Germany). Hydrochloric acid (37%, Merck KGaA, Darmstadt, Germany) was diluted to 0.1 mol L^−1^. The support materials for the SRF formulation were biosolids from a Colombian wastewater treatment plant of Medellín/WWTP San Fernando paper sludge (residual cellulose) and bentonite clay (sodium bentonite). Soil with a sandy texture (beach sand) previously washed with 0.1 mol L^−1^ HCl was employed in the soil-column assays.

Biosolids, paper sludge, and bentonite clay were characterized (results in g kg^−1^ dw or mg kg^−1^ dw (dw = dry-weight basis); see Table 2. For the physicochemical characterization, we employed established Colombian standards as follows: ash content was determined by gravimetric calcination at 650 °C (NTC 5167); oxidizable organic carbon (Cox) was quantified by the Walkley–Black dichromate wet-oxidation with a titrimetric endpoint (NTC 5167); total organic nitrogen was measured as Total Kjeldahl Nitrogen (TKN) by titrimetry (NTC 370); total phosphorus was analyzed after aqua regia digestion (HCl/HNO_3_) by UV–Vis spectrophotometry via the phosphomolybdovanadate complex (NTC 234); cation-exchange capacity (CEC) was determined volumetrically using ammonium acetate at pH 7.0 (NTC 5167); electrical conductivity (EC) was measured potentiometrically in a 1:100 extract (NTC 5167); and metals (Cr, Cd, Cu, Pb, Ni, Zn, and K) were quantified following aqua regia digestion by atomic-absorption spectrophotometry (NTC 5167). Microbiological safety of biosolids (*E. coli*, *Salmonella* spp., helminth eggs) followed NTC 5167 procedures and was benchmarked to EU 2019/1009 and NTC 5167:2011 limits.

To isolate carrier effects, matrix-only pellets (no fertilizer) with the same geometry/mass as SRF pellets were prepared: (i) FOMI-carrier only (biosolid: cellulose = 3:1; 1.68 g dw) and (ii) FOMII-carrier only (biosolid: clay = 1:1; 1.68 g dw). These controls were run in parallel in soil columns and dissolution vessels to quantify background N, P, and K release.

SRFs (FOMI and FOMII) were dried at 50 °C for 24 h, ground, and sieved (mesh #100). Pellets (1.68 g) were produced by extrusion–spherization [44,45], then analyzed physiochemically and microbiologically to guarantee sanitization (methods per NTC 5167; compliance checked against EU 2019/1009). Physicochemical tests included pH, Electric conductivity (EC), oxidizable organic carbon (C_ox_), TKN, total P, total K, bulk density, and the kinetics of nutrient release from the pellets into water. Microbiological tests included *E. coli*, *Salmonella* spp., and helminth eggs. See Table 2 for all abbreviations and symbols.

### 2.2. Occluded Fertilizer Formulation

The functionalized organic matrices (FOM) were prepared using the support materials, dried at 50 °C for 72 h, ground, and sieved through a mesh #100. The formulation with biosolid and sludge paper (biosolid and cellulose) in a 3:1 ratio was named FOMI. The formulation with biosolids and clay in a 1:1 ratio was named FOMII. Fertilizers were occluded into both matrices in a 50:50 ratio. The N, P, and K percentages were calculated using Equations (1)–(3) [46], where the endogenous N and P in biosolids were included in the mass balance.

(1) $$\;\;\;\%N=\frac{\left(W\;urea\times \%N\;urea+W\;biosolid\times \%\;N\;biosolid\right)}{W\;total}$$

(2) $$\%P=\frac{\left(W\;DAP\times \%P\;in\;DAP+W\;biosolid\times \%P\;in\;biosolid\right)}{W\;total}$$

(3) $$\%K=\frac{\left(W\;DAP\times \%P\;in\;DAP+W\;biosolid\times \%P\;in\;biosolid\right)}{W\;total}$$

where *W* means weight in grams.

The SRFs, FOMI and FOMII, were dried at 50 °C for 24 h, ground, and sieved through mesh # 100. Pellets of 1.68 g of FOMI and FOMII were prepared by the extrusion–spherization method [45,46] and subsequently analyzed physiochemically and microbiologically NTC 5167 [41], to guarantee the sanitization process.

### 2.3. Nutrient Release in Soil Columns

Nutrient release was evaluated using soil without fertilizer, soil fertilized with mineral fertilizer, soil amended with FOMI, and soil amended with FOMII.

Soil column assays were carried out using the incubation lysimeter methodology [47]. 2000 g of previously washed (0.1 mol L^−1^ HCl; Merck KGaA—Darmstadt, Germany) and sieved beach sand were placed in cylindrical PVC columns (ID 10 cm; height 30 cm; bottom mesh 0.5 mm). Columns were moistened to allow leachate percolation. Two 1.68 g SRF tablets were introduced at a 2.0 cm depth. 50 mL of water was sprayed daily (simulated rainfall 6.1 mm d^−1^). Leachate was collected daily for 30 d, stored at 4 °C, and analyzed for N, P, and K. Washed-sand blanks and matrix-only controls were run; background was not detected and subtracted from the time series.

**Figure 1:**
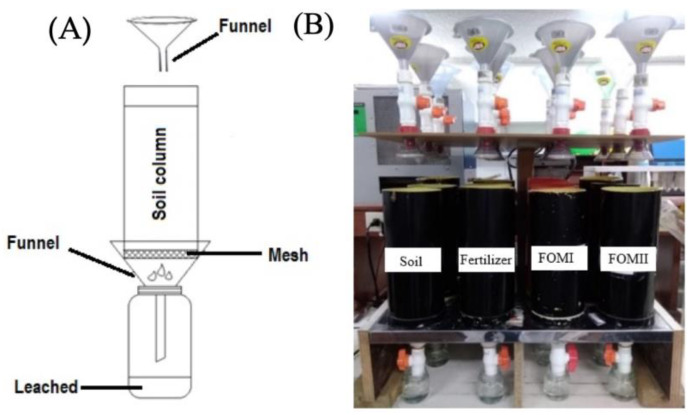
Lysimeter system. (**A**) Scheme. (**B**) Picture of real system.

The leaching process was repeated daily for 30 days, and the solution that percolated through the soil columns was collected and stored at 4 °C until analysis. Figure 1 shows the leach system. An analysis of the water percolated by the soil columns was also carried out to ensure that the NPK nutrient input only comes from the formulations.

### 2.4. Nutrient Release in Water

Prior to dosing any pellets, columns packed with the washed sand only were leached daily until background concentrations of N, P, and K were not detected (see QA/QC below). Final rinse waters from the acid-wash of sand were also analyzed to confirm the absence of residual N, P, and K. All experimental leachate time series were blank corrected using the background of the corresponding column.

The paddle dissolution method was used to conduct the slow-release tests in water utilizing a dissolver equipped with 1 L vessels and a mechanical stirrer for each vessel [48]. The working conditions were 1 tablet of 1.68 g per 500 mL of deionized water, 25 °C, and 100 rpm. The dissolution of nutrients from the pellet to the aqueous phase was determined by measuring the accumulated concentration of the fertilizer in terms of N, P, and K released, taking 15 mL aliquots at intervals of 10 min for 3 h. Potassium was determined by atomic absorption, P by the ammonium molybdovanadate method, and N by the Kjeldahl method.

Table 1 shows the different formulations evaluated in soil columns and in water. All assays were conducted in triplicate, and the ratio of nutrients was 10-10-10. In addition to Table 1, two matrix-only controls were included: (a) FOMI-carrier only, 2 pellets × 1.68 g dw per soil column (or 1 pellet per 500 mL in water); (b) FOMII-carrier only, same dosing. These controls allow verifying any intrinsic N, P, and K contribution from carriers.

### 2.5. Analytical Methods and QA/QC

All analyses were carried out in triplicate (n = 3) unless stated otherwise; results are reported as mean ± SD. Microbiological safety. *E. coli*, *Salmonella* spp., and helminth eggs followed NTC 5167 procedures and were benchmarked against EU 2019/1009 and NTC 5167 limits; results are summarized in Table 2 and Table 3. Drying steps were performed in a forced-air convection oven at the indicated set-points (e.g., 50 °C for 72 h for carrier preparation and 50 °C for 24 h for pellets).

### 2.6. Statistical Analyses

Assays in soil columns and water were analyzed using the Statgraphics program through ANOVA assay, to evaluate the differences between FOMI and FOMII parameters were evaluated using the Sigma Plot 12.5 program.

Time-course leaching and dissolution data were modeled with non-linear least squares to zero-order, first-order, and Higuchi release models; best-fit models were selected using AIC and extra-sum-of-squares F-tests. Where appropriate, repeated-measures ANOVA (treatment × time) was applied; assumptions of normality and homoscedasticity were checked with Shapiro–Wilk and Levene tests, respectively. If assumptions were violated, data were Box–Cox transformed or analyzed with Kruskal–Wallis followed by Dunn–Bonferroni post hoc tests. Between-treatment comparisons at specific times used one-way ANOVA with Tukey HSD (α = 0.05). Effect sizes (η^2^) and 95% confidence intervals are reported. Correlations between carrier properties (e.g., OM, CEC) and release metrics (e.g., t_50_, cumulative % at day 30) were assessed by Pearson/Spearman as appropriate.

## 3. Results and Discussion

### 3.1. Characterization of the Soil and Supporting Material

The physicochemical and microbiological parameters obtained from the analysis of the soil and supporting material are presented in Table 2. All analytical results in Table 2 are now reported as mean ± SD (n = 3) on a dry-weight basis; when applicable, the 95% CI is shown in parentheses. Metals are expressed as mg·kg^−1^ dw. “nd” indicates not detected. As discussed in the text, biosolids exhibit appreciable N (≈2.46% ± 0.88) and P_2_O_5_ (≈2.10% ± 1.29), relevant for calculating SRF nutrient load; sandy soil shows negligible NPK, confirming that nutrients are detected in leachates derived from the formulations. Microbiological criteria complied with NTC 5167/EU 2019/1009 (See Table 2).

The biosolids from the San Fernando WWTP have undergone a thickening, dehydration, drying, and anaerobic digestion process, with a retention time of 16 days and a temperature of 36 degrees Celsius, thereby reducing pathogens, fermentation power, and vector attraction capacity. The physicochemical analysis showed that the biosolids have nitrogen and phosphorus percentages of 2.46 and 2.10, respectively, which were considered for the SRF calculations, as observed in Equations (1)–(3). The CEC values contributed by the biosolid were 35.27 meq 100 g^−1^, complying with the minimum requirement of 30 meq 100 g^−1^ [41]. In addition, together with cellulose, they show a contribution of oxidizable organic carbon of 14.64% and 22.90%, respectively, reported as % by ISO 14235 [49]. According to the Colombian technical standard NTC 5167 [41], organic fertilizers must contain ≥15% contribution of oxidizable organic carbon. In this context, the value of 22.90% clearly meets the requirement. While the value of 14.64% is nominally below the threshold, it is still considered acceptable because its range of uncertainty (14.64 ± 2.22%) includes the 15% minimum threshold. It is essential to highlight that NTC 5167/04 applies strictly to the final commercial fertilizer product. Nevertheless, as these materials serve as raw materials (inputs) for fertilizer manufacturing, the fact that they already provide a carbon content that is at or near compliance provides a strong baseline and confirms their suitability as high-quality starting components.

This organic matter forms oxidized complexes, carboxylates (-COO^−^), which increase the CEC and together with the hydroxyl ions (OH^−^), present in cellulose, improves the CRA, which can adsorb exchangeable ions at the solid–liquid interface, thus trying to increase the concentration and volume of nutrients in the diffuse double layer, thus reducing the loss of nutrients in soil solution, by leaching and/or volatilization, reported in other studies [27,28].

The CRA values for biosolids and cellulose were 112.50 and 264, respectively, being higher than 100%, complying with the same standard, which establishes that their own weight must be minimal. It was also observed that heavy metals were within the limits allowed by law (<400 ppm) [41]. With the values observed in the previous variables, the efficiency of the SRF is largely guaranteed. Furthermore, as can be seen in Table 1, the NPK values present in sandy soil are insignificant, ensuring that nutrients only come from the formulations.

### 3.2. Characterization of the Occluded Fertilizers

Table 3 shows the physicochemical and microbiological parameters of the occluded fertilizers FOMI and FOMII. Also presents pH, OM, CEC, WRC, OC, and microbial counts as mean ± SD (n = 3) with 95% CI in parentheses. The entries for total N, P_2_O_5_, and K_2_O in FOMI/FOMII correspond to the formulated 10–10–10 target; the measured totals (mean ± SD) are provided in Table 3, final three rows, to disclose analytical uncertainty explicitly. Cellulose-rich FOMI shows higher OM and WRC than FOMII, supporting a stronger water-uptake/swelling capacity and potential for nutrient retention via H-bonding and carboxylate complexation; FOMII presents slightly higher CEC, consistent with clay mineral negative charge (isomorphic substitution).

**Figure 2:**
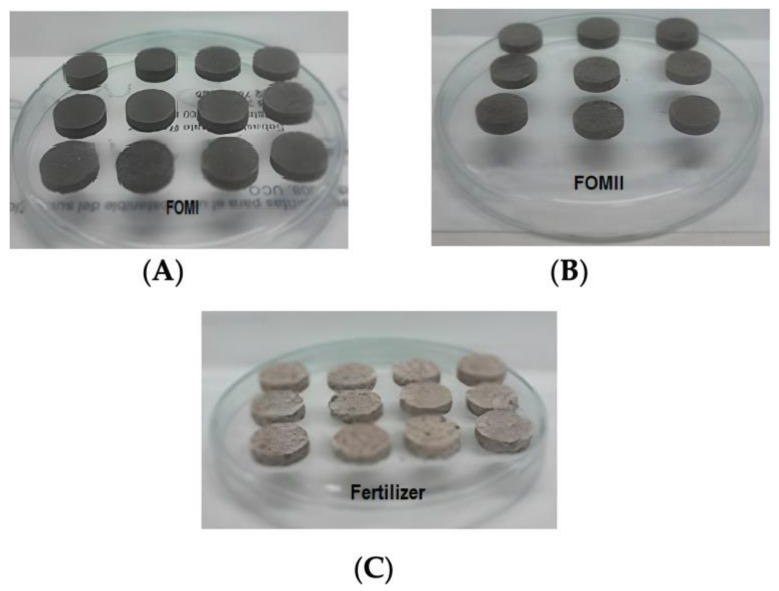
Pellets (**A**) Biosolid/cellulose (**B**) Biosolid/clay (**C**) Fertilizer.

The pH values were acceptable according to NTC 5167. Enterobacteria decreased during the occlusion process relative to the matrix, from 1 × 10^5^ CFU g^−1^ to 9.5 × 10^2^ CFU g^−1^. As reported in [50], this reduction is consistent with crenation and growth inhibition driven by the osmotic pressure exerted by N, P, and K salts. CEC values for FOMII were slightly higher, likely due to silicate groups in the clay (SiO_4_)^4−^, whereas the higher OM and WRC of FOMI support the formation of oxidized complexes and hydrogen bonds, favoring a steadier, controlled nutrient supply [25]. In contrast, the mineral fertilizer—by formulation—does not contribute to OM or WRC and typically shows burst release in early periods, as also reported elsewhere [25]. Representative pellets of the three treatments are shown in Figure 2A–C).

### 3.3. Evaluation of the Release of Nutrients in the Water

**Figure 3:**
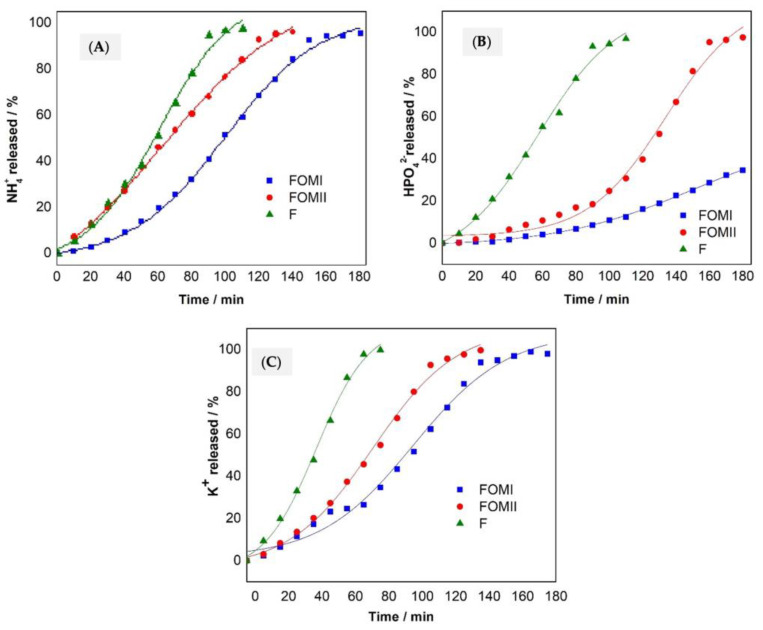
Nutrients released in water: (**A**) Nitrogen as NH_4_^+^, (**B**) Phosphorus as HPO_4_^2^−, (**C**) Potassium as K^+^.

Release of macronutrients (N, P, and K) as NH_4_^+^, HPO_4_^2−^, and K^+^ from the tablets to the aqueous medium in the laboratory conditions is shown in Figure 3. Error bars in Figure 3A–C display SD (n = 3); shaded bands show 95% CI of the best-fit curve.

- FOMI delivered a steadier nutrient supply, releasing <25% of N, P, and K during the first hour; the curves remain sublinear through 180 min, indicating diffusion/swelling control.
- FOMII released ≈50% N and >35% K in the first hour; P release was similar to FOMI early on but accelerated thereafter.
- Mineral fertilizer (F) showed the expected burst: ~50% of N and P in the first hour, and ~100% by 80–100 min (K completed by ~80 min).

The gentler slopes for FOMI reflect higher WRC and OM (Table 3), which promote swelling and H-bond networks that retain water and dissolved ions, dampening concentration gradients and reducing instantaneous flux versus F and FOMII.

The FOMI formulation showed a steadier supply of nutrients N, P, and K in water, releasing less than 25% in the first assay hour. FOMII lost about 50% of nitrogen and more than 35% of potassium in the same period. The phosphorous behavior was similar in both FOMI and FOMII. Inorganic fertilizer released 50% of the nutrients in the first hour, and released all its nutrient content within 100 min of testing, in the case of N and P, and at 80 min for K, unlike the FOMI, which continued to release nutrients until the end of the test. The high solubility of the inorganic fertilizer in water causes rapid dissolution of tablets, accelerating the release of nutrients [51].

The controlled release in FOMI is due to the cellulose in the paper sludge. The CEC and WRC increased, capturing and exchanging cations from inorganic fertilizer, avoiding leaching losses [52]. Cellulose can form hydrogen bonds with water, acts as a retainer polymer, increases the swelling capacity, and captures water and nutrients [25]. The presence of cellulose helps preserve and delay the delivery of nutrients, regulating their release and minimizing their exposure.

Equation (4) can be used to analyze the controlled release behavior of occluded fertilizers according to the Korsmeyer–Peppas method [53].

(4) M_t/M_∞=kt^n

where M_t/M_∞ is the fraction of released fertilizer at time *t*, M_∞ is the amount of released fertilizer after infinite time, k is the kinetic constant and involves the structural and geometric characteristics of the tablet, and n is the diffusional exponent, which depends on the release mechanism. A value of n ≤ 0.5 means that Fickian diffusion controls the release mechanism of fertilizer. Instead, an n value equal to one (n = 1) indicates an anomalous transport phenomenon (kinetic order 0). The n values major to one (n > 1) mean strange anomalous diffusion (not Fickian) and can include different phenomena, such as diffusion and swelling [54]. Equation (5) shows the linearized form of Equation (4).

(5) LogM_t/M_∞=log(k)+nlog(t)

### 3.4. Kinetic Modeling in Water

**Figure 4:**
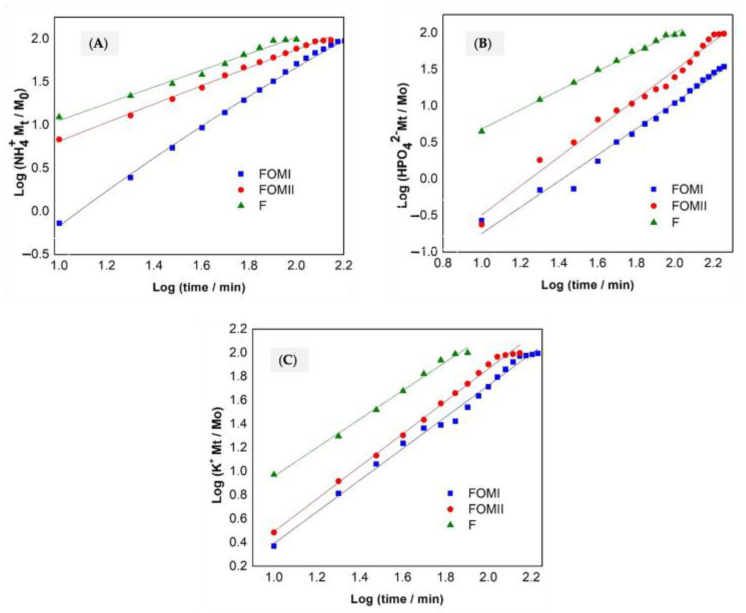
Korsmeyer–Peppas model adjusts (**A**) Nitrogen as NH_4_^+^, (**B**) Phosphorus as HPO_4_^2−^, and (**C**) Potassium as K^+^.

Figure 4 shows the adjustment to the Korsmeyer–Peppas model, and Figure 4**.** Korsmeyer–Peppas model adjusts (**A**) Nitrogen as NH_4_^+^, (**B**) Phosphorus as HPO_4_^2−^, and (**C**) Potassium as K^+^.

Table 4 shows the parameters obtained for the different treatments. The log–log plots (Figure 4A–C) fit the Korsmeyer–Peppas model with R^2^ ≈ 0.98–0.996. Table 4 now reports k and n as estimates ± SE (95% CI) for each ion and treatment.

- Both FOMI and FOMII yield n > 1 (super case II), consistent with swelling/relaxation-controlled transport in hydrated matrices; k values are lowest for FOMI, indicating the slowest apparent release rate.
- F exhibits the largest k and near-unit or sub-unit n, consistent with rapid erosion/dissolution and minimal matrix control (burst effect).
- Taken together, the kinetic parameters confirm that matrix composition (cellulose vs. clay) governs the extent of swelling and diffusional resistance, explaining the rank order F > FOMII > FOMI in release rate.

Both FOMI and FOMII formulations presented values of n greater than 1, which correspond to non-Fikian linear release kinetics characterized by an increase in the water absorption rate in the matrix. This type of diffusion cannot explain the nutrient behavior in a complex medium because of the solid phase in the nutrient diffusion. The kind of matrix controlling the release rate. In our case, we can think of a hydrogel model in which the fertilizer is in the core, and the water diffuses and begins to solubilize the fertilizer, increasing the osmotic pressure in the system and causing the nutrients to come out in favor of the concentration gradient [53,55]. The analysis of Table 4 allows us to observe that the FOMI showed k values lower than FOMII and the fertilizer, suggesting that the rate of nutrients released in FOMI is more controlled. Cellulose hydroxyl groups (OH−) can form hydrogen bonds with water, increasing the affinity of the matrix with the medium and its swelling and favoring the retention of water and nutrients dissolved in it, explaining the phenomenon [44,52].

Fertilizers do not have a release mechanism because the tables are eroded quickly. The k values were higher in relation to FOMI and FOMII, showing that fertilizer presented a fast, uncontrolled initial discharge, known as the “explosion effect” [25]. All correlation coefficients were superior to 0.98, indicating that the empirical Korsmeyer–Peppas model describes the release process and can be used to explain the nutrient behavior in an aqueous solution.

Figure 3A–C show the plots of release fractions of fertilizer for Korsmeyer–Peppas in water. By plotting the log of the fraction of nutrients released against the log of t, the slope and intercept values corresponding to n and k, respectively, were obtained. The results are shown in Table 3. The MOFI and MOFII formulations presented n values greater than 1.0, which corresponds to a non-Fikian linear release kinetics super case II characterized by an increase in the rate of water absorption by the matrix [56]. Furthermore, as can be seen from Table 3, all correlation coefficients R^2^ were close to 0.99, showing that the Korsmeyer–Peppas empirical model adequately describes the release process and can be used to explain the behavior of nutrients in aqueous solution, reported in drug release studies [57].

### 3.5. Evaluation of the Release of Nutrients in Soil Columns

**Figure 5:**
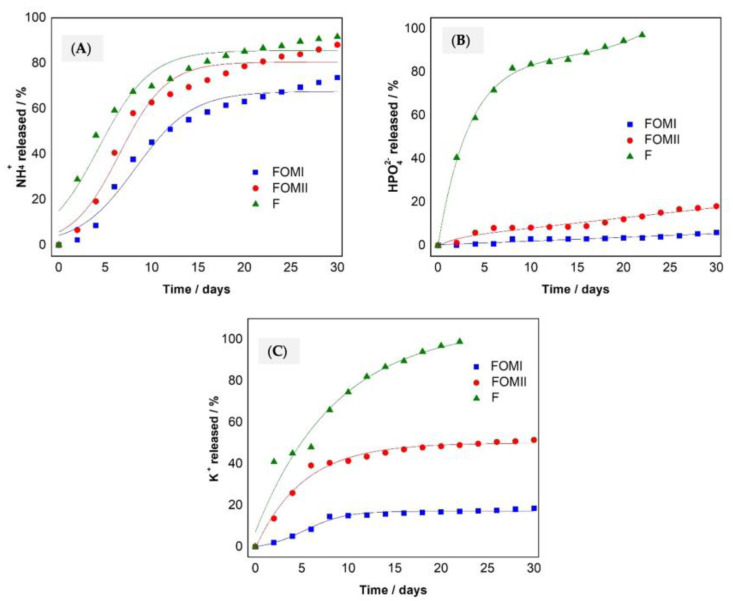
Release of nutrients in soil columns (**A**) Nitrogen as NH4^+^, (**B**) Phosphorus as HPO_4_^2^−, and (**C**) Potassium as K^+^.

Figure 5 shows the percentage of release of macronutrients N, P, and K, expressed as NH_4_^+^, HPO_4_^2−^, and K^+^, from the tablets to soil columns in the laboratory conditions. Results in soil (beach sand) without fertilizer (blank) were undetectable. Error bars represent SD (n = 3); curves include 95% CI bands from repeated-measures fits.

- According to European Committee for Standardization (CEN) SRF criteria, FOMI qualifies as SRF (≤15% release at 24 h; ≤75% at 28 d). FOMI/FOMII both released <15% N at 24–48 h (FOMI ~2.2%, FOMII ~6.6% at 48 h). By day 28, N release reached ~71.5% (FOMI) and ~85.9% (FOMII), while F exceeded 90%.
- For P and K, FOMI consistently released less than FOMII (end-point P: ~6% vs. 18%; end-point K: ~18.5% vs. 51.4%), and F exhausted both nutrients by ~22–23 days.

The leachates from the washed-sand blanks did not detect values for N, P, or K during the first 10 days, confirming no prior nutrient contribution from the matrix/packing.

The superior retention in FOMI is consistent with its higher OM and WRC (Table 3), enabling a hydrogel-like behavior that stores water, increases diffuse double-layer thickness, and slows ion transport to percolate.

The European Committee for Standardization (CEN) considers a fertilizer an SRF if it does not release more than 15% of the nutrient during the first 24 h and does not release 75% in 28 days [8]. So, considering the quantities released of N, P, and K, FOMI has an SRF behavior. FOMI and FOMII only showed differences in the nitrogen content at 28 days; during the first 24 h, less than 15% of N was released for FOMI and FOMII. Even in 48 h, the N release percentages were less than 15%, with only 2.18% and 6.56% for FOMI and FOMII, respectively. The release percentages on day 28 were, respectively, 71.5% and 85.9% for FOMI and FOMII. Inorganic fertilizer released more than 29% of its nutrients during the first 24 h and 90% on day 28.

In the case of P and K releases, the behaviors followed the same trend, where FOMI showed lower levels of nutrient release than FOMII. In the case of phosphorus, a nutrient release of 6% and 18%, respectively, could be observed at the end of the process, unlike the fertilizer, which released all the nutrients after 23 days of testing. In the case of potassium, the releases were 18.5% and 51.4%, respectively, and the fertilizer released all the nutrients after 22 days of testing.

The water percolated by the sandy soil columns was collected during the first ten days of the trial, and NPK analysis was performed to determine if there was a previous contribution of these nutrients to the matrices. The analysis of the water percolated by the soil columns is shown in Table 5.

As can be seen in Table 5, the results of the water collected through the sandy soil columns did not show significant amounts of NPK, which guarantees that all the nutrients come from the initial formulations.

Results show that the occlusion of fertilizers F, (NH_4_)_2_HPO_4_, (NH_2_)_2_CO, and KCl in the evaluated organic matrices had a rate-controlled release during the 30 days of the assay, taking more than three times as long to deliver 50% of the nutrients compared with the inorganic fertilizer. An ANOVA analysis showed significant differences (*p* ≤ 0.05) between the release percentages of nutrients in FOMI, FOMII, and F. As can be seen, the FOMI met the criteria to be considered an SRF and additionally had higher retention of nutrients, with N, P, and K concentrations superior to 25% after 28 days, since this formulation does not release more than 15% of nutrients during the first 24 h and no more than 75% after 28 days.

First-order kinetics can moderate the release of nutrients, in which the rate of nutrient release is proportional to the relation between the concentration at a defined time and the initial concentration of the fertilizer, according to Equation (6) [58].

(6) $$F=F_{0}e^{-kt}\;;\;\frac{\left[F\right]}{{\left[F\right]}_{0}}=e^{-kt}$$

where *F* = concentration of fertilizer in a time *t*, *F*_0_ = initial concentration of the fertilizer, and *k* = kinetic constant. Shows the adjusted parameters to first-order kinetics obtained using Equation (7).

The correlation coefficients were in the range of 0.95–0.99, showing that the process of nutrients being released from the organic matrix to a complex medium like the soil can be described by first-order kinetics. As for water assays, FOMI had smaller k values than the FOMII formulation and the inorganic fertilizer. A multiple-range test showed significant differences between the release constants for the nutrients. FOMII ‘s release nitrogen percentage exceeded the limits established for an SRF, indicating that FOMI has a lower rate of nutrient release and more controlled delivery. FOMI behavior can be explained by the presence of cellulose, which increases the CEC and WRC due to its higher content of OM. The functionalized cellulose favors the formation of oxidized complexes such as carboxylates (R-COO−) and hydrogen bonds due to the functional hydroxyl (OH^−^) groups, which, together with the carboxyl groups (-COOH), have been reported as responsible for the hydrophilic character of hydrogels [25]. FOMI behaves as a hydrogel, increasing its surface area and swelling capacity, retaining water quantities greater than its weight, and preserving and exchanging nutrients, reducing leaching losses [25,59,60].

### 3.6. Statistical Analyses

Comparisons between the different formulations with the Stratigraphic program and the analysis of variance (ANOVA), for the percentages of release of N, P, K, it could be observed in the case of nitrogen that the Biosolids/cellulose matrix compared with the fertilizer presents a value of *p* = 0.0148115; (*p* ≤ 0.05), which demonstrates the existence of statistically significant differences between the means of these two formulations. In the case of phosphorus and potassium, values of (*p* ≤ 0.05) were also observed, corresponding to *p* = 0.0007 and *p* = 0.0006, respectively.

All time-series were analyzed by repeated-measures ANOVA (treatment × time); when assumptions were violated, Box–Cox transformations or Kruskal–Wallis + Dunn–Bonferroni were used.

- In water (Figure 3 and Figure 4), k differed among treatments (*p* < 0.05), with FOMI < FOMII < F for N, P, and K.
- In soil (Figure 5), treatment effects were significant for all nutrients (*p* < 0.05); post hoc Tukey HSD showed FOMI vs. F and FOMI vs. FOMII differences at most time points.
- Where stated in the original text, ANOVA results (e.g., *p* = 0.015 for N in FOMI vs. F; *p* < 0.001 for P and K) are retained and now accompanied by effect sizes (η^2^) in the figure captions.

## 4. Conclusions

Occluding inorganic fertilizers within the biosolids–paper-sludge organic matrix (FOMI/NPK) increased the water-retention capacity (WRC) and cation-exchange capacity (CEC) while adding organic matter, yielding a more controlled nutrient release that meets CEN slow-release fertilizer (SRF) criteria. Microbiological compliance (NTC 5167/EU 2019/1009) indicates no environmental or health risk. Given its biodegradability, low cost, and use of residual streams, FOMI/NPK is a sustainable option for recovering soils degraded by intensive agriculture and excessive agrochemical use.

The biosolids/paper-sludge formulation (FOMI/NPK) is a promising carrier for controlled delivery of nutrients and potentially other actives (e.g., herbicides, pesticides). The de-inking/delignification of cellulose increases hydrophilicity and promotes swelling and intra/intermolecular H-bonding (−OH, −COOH), which stabilizes the matrix, enhances water and ion retention, and thereby dampens the initial burst and cumulative losses. This mechanistic behavior explains the improved WRC/CEC observed for FOMI relative to mineral fertilizer alone and to the bentonite-based alternative.

Kinetic analyses showed that the biosolids/cellulose matrix (FOMI) exhibits lower k values than both the biosolids/clay matrix (FOMII) and the mineral fertilizer, indicating a slower, diffusion-/swelling-controlled release that reduces leaching. This can be attributed to polymer water uptake and the capacity of cellulose −OH and −COOH groups to form hydrogen bonds and weak complexes with nutrient ions. Together with the washed-sand blanks and matrix-only controls (negligible background N, P, K), these results support that FOMI/cellulose/NPK can improve fertilizer use efficiency and conserve water in agricultural applications.

## Figures and Tables

**Table 1:** Formulations for the different assays in the soil and water columns.

Formulation	Assay in Soil Columns (2 Pellets with 1.68 g)	Assay in Water (1 Pellet with)
Mineral fertilizer (F), control	NPK (10-10-10) = 3.36 g/soil	NPK (10-10-10) = 1.68 g/500 mL
Organic matrix (FOMI)	50% fertilizer + 50% FOMI	% fertilizer + 50% FOMI
Organic matrix (FOMII)	50% fertilizer + 50% FOMII	% fertilizer + 50% FOMII
Soil without fertilizer (blank)	2 kg/soil column	-

**Table 2:** Physicochemical and microbiological parameters of the soil and supporting material.

Parameter	Sandy Soil	Biosolids	Cellulose	Bentonite Clay
Total nitrogen (N)%	0.067	2.46 ± 0.88	0.38 ± 0.50	ND
Total phosphorus (P_2_O_5_),%	ND	2.10 ± 1.29	0.46 ± 0.98	ND
Total potassium (K_2_O),%	0.0114	0.64 ± 0.65	ND	0.0117 ± 0.0001
Water retention capacity (WRC), %	20.57	112.50 ± 0.03	264 ± 0.08	26 ± 0.3
Cation exchange (CEC), meq 100 g^−1^	13.26	35.27 ± 2.03	9.31 ± 0.04	24.40 ± 0.5
Humidity (H), %	0.12	59.66 ± 0.58	55.60 ± 0.05	28.70 ± 0.07
pH	6.37	7.01 ± 0.48	7.73 ± 0.03	6.5 ± 0.17
Electric conductivity (EC), dS m^−1^	0,02	4.76 ± 0.17	0.02 ± 0.12	0.040 ± 0.9
Oxidizable Organic Carbon (C_ox_)%	2.01	14.64 ± 2.22	22.90 ± 1.20	ND
Ashes, %	94.95	64.01 ± 2.88	42 ± 0.03	ND
Density, g cm^−3^	1.72	0.48 ± 0.08	0.36 ± 0.008	2.62 ± 0.003
Microbiological parameter				
Mesophiles CFU g^−1^	ND	9.48 × 10^10^ ± 1.10 × 10^10^	1.3 × 10^9^	ND
Thermophiles CFU g^−1^	ND	4.52 × 10^8^ ± 1.29 × 10^9^	8.8 × 10^8^	ND
Enterobacteria CFU g^−1^	ND	1.0 × 10^5^ ± 2.07 × 10^5^	2.0 × 10^1^	ND
*Salmonella* sp. (absent in 25g)	ND	ND	ND	ND
Metals				
Na %	0.0082	0.17 ± 0.10	ND	0.040 ± 0.0001
CaO %	ND	3.78 ± 0.317	9.12 ± 0.02	0.068 ± 0.0002
MgO %	0.0073	0.56 ± 0.31	0.24 ± 0.04	0.120 ± 0.001
Zn, ppm	ND	0.39 ± 0.27	ND	0.00036 ± 0.0005
Cr, ppm	ND	381.57 ± 3.0	55.18 ± 0.35	ND
Cd, ppm	ND	2.72 ± 2.51	ND	ND
Pb, ppm	ND	36.98 ± 2.22	ND	ND
Ni, ppm	ND	149.38 ± 2.88	11.71 ± 0.77	ND
As, ppm	ND	0.18 ± 3.20	16.51 ± 0.67	ND

ND: not detected. All physicochemical and microbiological results are reported on a dry-weight basis. Source: GIEM Laboratory, UdeA.

**Table 3:** Physicochemical and microbiological parameters of the occluded fertilizers.

Parameter	FOMI	FOMII
pH	6.68 ± 0.09	6.57 ± 0.08
Organic carbon (OC), %	17.43 ± 0.12	10.0 ± 0.01
Cation exchange (CEC) meq 100 g^−1^	76.8 ± 0.06	82.9 ± 0.05
Water retention capacity (WRC)	205 ± 0.03	38.5 ± 0.02
Total nitrogen (N) (%)	10	10
Phosphorus, P_2_O_5_ (%)	10	10
Potassium, K_2_O	10	10
Enterobacteria CFU g^−1^	9.5 × 10^2^	2.2 ×10^2^
Mesophiles CFU g^−1^	2.5 ×10^6^	6.4 ×10^8^
Thermophiles CFU g^−1^	2.4 ×10^10^	7.5 ×10^8^

**Table 4:** Parameters of the Korsmeyer–Peppas model adjust.

Kinetic Model	Parameter	FOMI			FOMII			Fertilizer		
Korsmeyer–Peppas		NH_4_^+^	HPO_4_^2−^	K^+^	NH_4_^+^	HPO_4_^2−^	K^+^	NH_4_^+^	HPO_4_^2−^	K^+^
	n	1.753	1.753	1.333	1.055	1.980	1.373	0.964	1.315	1.204
	K	0.014	0.003	0.115	0.579	0.004	0.131	1.226	0.234	0.568
$\frac{M_{t}}{M_{\infty }}=Kt^{n}$	R^2^	0.994	0.982	0.992	0.996	0.985	0.996	0.988	0.995	0.568

**Table 5:** The analysis of the water percolated by the soil columns.

Day	% N	% P	% K
1	−0.0002225 ± 0.00012	−0.0000864 ± 0.00007	0.0002490 ± 0.00018
2	−0.0006044 ±0.00099	0.0067099 ± 0.00010	0.0002745 ± 0.00021
3	−0.0004973 ± 0.00021	0.0018617 ± 0.00012	0.0000761 ± 0.00020
4	−0.0001310 ± 0.00024	0.0030530 ± 0.00010	0.0001516
5	−0.000515568	0.00543551	-
6	−0.000160714	0.00266179 ± 0.00020	0.0013831 ± 0.00011
7	−0.000964286	0.0028391 ± 0.000019	0.0002936 ± 0.00010
8	−0.000717033 ± 0.00013	0.0015980 ± 0.000271	-
9	−0.000465201 ± 0.00020	0.00181959	-
10	−0.000304945 ± 0.00031	0.00221853 ± 0.00024	-

## Data Availability

No new data were created or analyzed in this study. Data sharing is not applicable to this article.
